# Development and evolution of the MHAUS cognitive aid for malignant hyperthermia

**DOI:** 10.1186/1471-2253-14-S1-A25

**Published:** 2014-08-18

**Authors:** Teeda Pinyavat, Cynthia Wong, Henry Rosenberg

**Affiliations:** 1 Department of Anesthesiology, Columbia University College of Physicians and Surgeons, New York City, NY 10032, USA; 2 Department of Anesthesiology, Northwestern University Feinberg School of Medicine, Chicago, IL 60611, USA; 3 Department of Medical Education and Clinical Research, Saint Barnabas Medical Center, Livingston, NJ 07039, USA

## Background

Cognitive aids help the stressed practitioner in an emergency setting carry out complex tasks. Ideally, they ensure the completion of key steps and prevent unnecessary ones. Malignant hyperthermia is an emergency event that lends itself to the use of a cognitive aid. Experts associated with the Malignant Hyperthermia Association (MHAUS) first developed a cognitive aid in the form of a “poster” in the mid-1980s.

## Methods and results

MH experts based the “poster” on review of the current literature, personal experience, and experience gained from the MH Hotline. The first four versions (1991, 1993, 1995, and 1998) were formatted as a list of steps. In the first ten years, changes were mainly to content rather than design. For example, dantrolene administration was prioritized - Step 1 “GET HELP.GET DANTROLENE,” changing the anesthesia circuit tubing and CO_2_ absorbent were no longer recommended, and specific dosages were given for insulin and glucose for the treatment of hyperkalemia.

Released in 2003, the fifth version of the MH poster had a completely different format with three separate sections for diagnosis (including masseter spasm and sudden cardiac arrest in a child), acute treatment, and post acute care. The sentences were converted to bullet points and formatted in columns to make reading easier. Bolded text was also used to highlight common errors, such as failing to use sterile water to dilute the dantrolene, and the avoidance of calcium channel blockers. The next two versions (2005 and 2008) retained this format with added information on myoglobinuria and the maintenance of adequate urine output.

The latest version (2011), has a dramatically different look. The most notable changes are the use of color, pictures, and an algorithm style rather than bullet-point steps.

Over 20,000 posters have been distributed over past several decades. The use of the posters/algorithm has been incorporated into simulation-based scenarios involving MH; the posters have has been translated into different languages, and modified for inclusion in the Stanford Emergency Manual.

## Conclusions

The MHAUS MH treatment poster was one of the, if not the first cognitive aid to be used in anesthesia practice. It has evolved from a simple list of steps to a visually appealing and easy to follow algorithm that incorporates all key treatment elements and common diagnostic questions. Although it is impossible to measure how the “poster” has influenced the recognition and treatment of MH, the fact that it is widely distributed and continues to be in demand attests to its probable effectiveness.

